# Supplementary material for: ADAM10-Mediated Cleavage of ICAM-1 Is Involved in Neutrophil Transendothelial Migration
Source: Cells. 2021 Jan 25;10(2):232. doi: 10.3390/cells10020232 (PMC7911467; doi:10.3390/cells10020232)
Supplement: Supplementary file 1 [file cells-10-00232-s001.pdf]

FIGURE S1

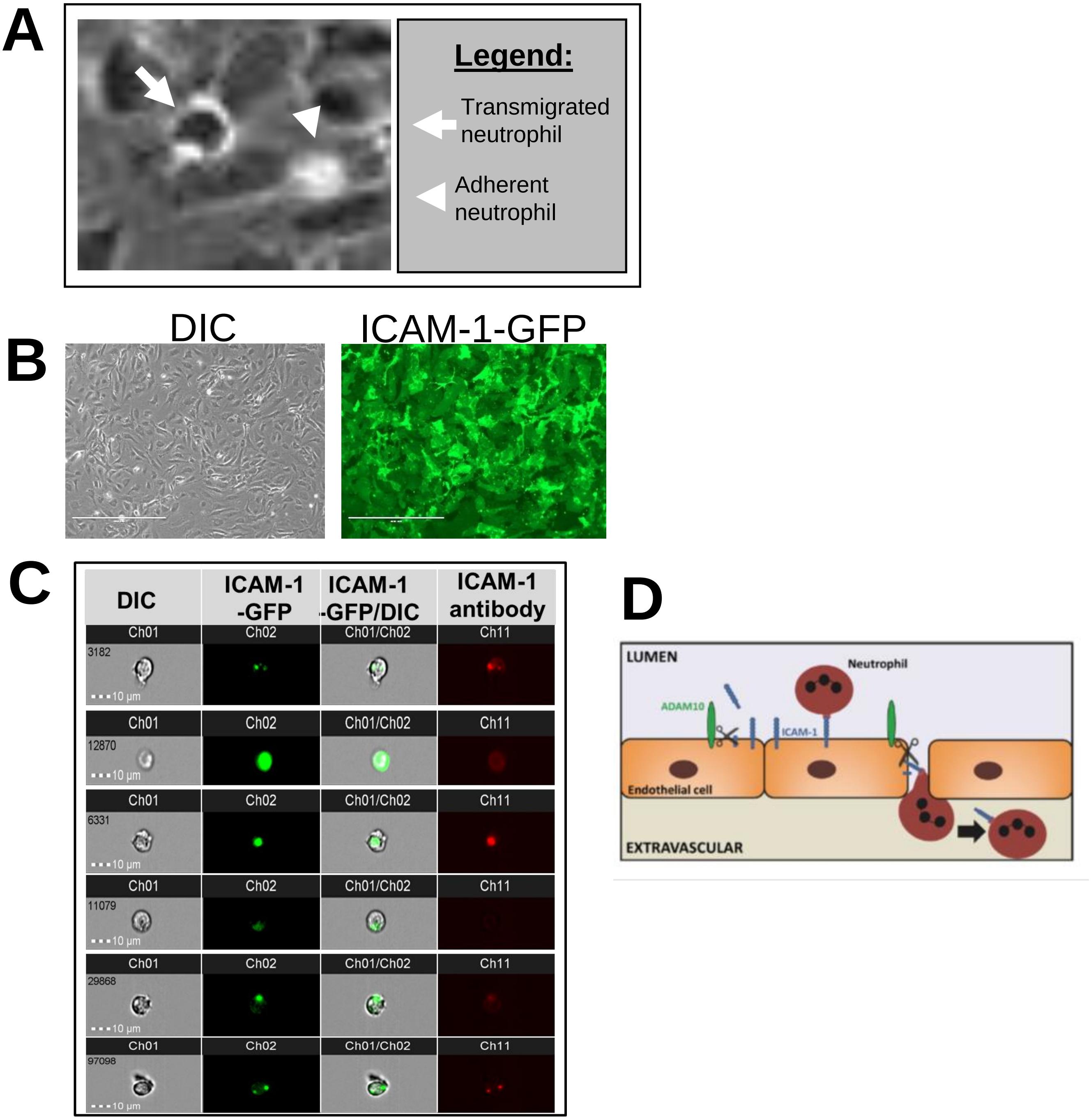

Figure S1. (**A**) ECs were applied to flow in the TEM flow chamber and perfused with primary human neutrophils. Phenotype of the neutrophils detected with DIC microscopy discriminates between a crawling neutrophil (bright dot phenotype, arrow) and a transmigrated neutrophil (dark-grey phenotype, arrowhead). (**B**) ECs were transfected with ICAM-GFP as indicated. >95% of all endothelial cells were transfected. (**C**) ImageStream example of positive neutrophils, as indicated. Neutrophils were additionally incubated with an ICAM-1-antibody (red). (**D**) Schematic overview of proposed mechanism by which shedding of the extracellular domain of ICAM-1 by endothelial ADAM10 is involved in neutrophil diapedesis.
